# Glioblastoma Prognosis and Therapeutic Response Predicted by a Cancer‐Associated Fibroblasts Risk Score

**DOI:** 10.1155/mi/4342537

**Published:** 2025-12-28

**Authors:** Hongyi Zhou, Xi Yang, Wen Zhao, Yingqi Huang, Zhixiang Zhang, Jincheng Jiang, Xinchen Jiang, Bingxuan Ren, Kaixia Yang

**Affiliations:** ^1^ Department of Anus and Intestine Surgery, The Affiliated Lihuili Hospital of Ningbo University, Ningbo University, Ningbo, Zhejiang, 315040, China, nbu.edu.cn; ^2^ Department of Neurology, The Affiliated Lihuili Hospital of Ningbo University, Ningbo University, Ningbo, Zhejiang, 315040, China, nbu.edu.cn; ^3^ Wenzhou Medical University, Wenzhou, Zhejiang, 325027, China, wmu.edu.cn

**Keywords:** cancer-associated fibroblasts, glioblastoma, immunotherapy, prognosis, tumor immune microenvironment

## Abstract

**Background:**

Cancer‐associated fibroblasts (CAFs), as a key component of the tumor microenvironment, have not been systematically elucidated in glioblastoma (GBM). Our study aims to develop a prognostic model integrating CAFs‐related features, with the goal of providing new insights for precise stratification and optimized treatment strategies for GBM patients.

**Methods:**

Utilizing GBM‐related data from reputable public databases, we utilized the Seurat package in R to analyze single‐cell RNA sequencing (scRNA‐seq) data for the characterization of CAFs in GBM. We identified CAFs phenotypes and screened for key CAFs‐related genes significantly associated with patient prognosis. Using regression analysis, we constructed a CAFs‐based risk score, which was subsequently validated in multiple independent cohorts. A nomogram integrating the risk score and clinicopathological features was also developed. Furthermore, we systematically evaluated the prognostic and therapeutic relevance of the model in GBM patients through multi‐dimensional analyses, including gene mutation profiling, pathway enrichment analysis, immune infiltration, immunotherapy response, and drug sensitivity analysis.

**Results:**

A total of six CAFs‐related genes (FAM241B, LSM2, IGFBP2, LOXL1, OSMR, and STOX1) were identified as significantly associated with GBM prognosis. We used it to construct the CAFs‐based risk score model, which demonstrated robust prognostic performance across multiple cohorts and served as an independent predictor of overall survival in GBM patients, efficiently categorizing groups into high and low risk. By integrating clinical features, the nomogram model significantly increased predictive accuracy and reliability. Analytical results indicated a statistically significant association between the computed risk score and the level of immune cell infiltration. Furthermore, the established prognostic model exhibited robust efficacy in predicting patient outcomes following conventional targeted treatments as well as immunotherapeutic interventions.

**Conclusions:**

This study introduces a GBM risk profiling framework and accompanying nomogram, offering exceptional accuracy in prognostic prediction for GBM. The framework and nomogram provide valuable insights into the roles of CAFs and key genes in GBM progression and immunity, and extend beyond classification by offering promising avenues for deciphering tumor mutations, mapping immune landscapes, refining drug predictions, and forecasting the efficacy of immunotherapeutic interventions. These findings have the potential to significantly improve personalized treatment strategies and patient outcomes.

## 1. Introduction

Glioblastoma (GBM), which is classified by the World Health Organization (WHO) as a Grade IV malignant glioma, with the highest degree of malignancy and represents the most common primary malignant tumor of the central nervous system in adults [1]. GBM originates from glial cells and exhibits extremely high invasiveness and heterogeneity. Clinically, it often manifests as rapid proliferation, diffuse infiltrative growth patterns, and is associated with a very high risk of postoperative recurrence [2–4]. Therefore, improving the treatment and prognosis of GBM patients remains a major unresolved challenge in the field of neuro‐oncology [5].

Presently, the treatment of GBM is fraught with challenges, particularly due to the immunosuppressive tumor microenvironment (TME), the formidable blood‐brain barrier, and pervasive heterogeneity at both molecular and cellular levels [6]. The complexity of this scenario is heightened by the diverse molecular makeup of GBM and the array of cytokines its cells secrete, which pose considerable challenges. Conventional targeted therapies demonstrate limited efficacy, while immunotherapy has emerged as a promising novel strategy in recent years, offering new hope for the treatment of GBM [6, 7]. This cutting‐edge technique harnesses the body’s own immune defenses to target and remove cancer cells and tumor tissue. Among the myriad immunotherapy strategies, four have garnered substantial attention and research momentum for their potential in treating GBM: immune checkpoint blockades (ICBs), adoptive cell therapies, therapeutic vaccines, and oncolytic virus therapies (OV therapy). ICBs represented by Programmed Cell Death Ligand 1 (PD‐1/PD‐L1) monoclonal antibodies have achieved breakthroughs in certain clinical contexts. They effectively reverse PD‐1/PD‐L1‐mediated immunosuppression and significantly enhance antitumor responses, showing potential for prolonging survival in patients with recurrent or resected GBM [8]. However, trials involving adjuvant PD‐1/PD‐L1 therapy in newly diagnosed GBM patients have not achieved the desired outcomes [9, 10], prompting further investigation into the underlying mechanisms.

As insights into the complex TME expand, the diverse compositions of GBM microenvironments across individual patients have gained greater attention, underscoring their crucial role in shaping prognosis. The TME provides essential nutrients for tumor growth, promotes tumor cell proliferation and metastasis, facilitates immune evasion, and contributes to tumor heterogeneity [11–13], thus representing a major barrier to the effective treatment of GBM. Among these components, CAFs as a central stromal cell type in the TME, play a prominent role in tumor initiation, proliferation, metastasis, induction of heterogeneity, treatment resistance, and poor prognosis [14, 15]. Because of these functions, CAFs have been seen as tumor‐promoting and are regarded as promising therapeutic targets. Notably, CAFs are highly heterogeneous, and their characteristics and interactions with other cellular components may change dynamically during cancer progression [16, 17]. Some research has indicated that CAFs are present in GBM and their potential to promote tumors [18], a systematic investigation into their cellular diversity, functional roles, and clinical prognosis associations is still missing. Thus, thoroughly examining the diversity and clinical importance of CAFs in GBM may help elucidate their potential pathogenic mechanisms and offer a theoretical foundation for creating new therapeutic approaches.

Consequently, this study develops a risk prediction model with CAFs data in GBM to forecast patient outcomes. Transcriptome and single‐cell RNA sequencing (scRNA‐seq) datasets were collected from reliable public repositories and underwent detailed examination. This process led to the identification of distinct CAFs subclusters and the development of a risk stratification algorithm, whose independent prognostic value was validated using GBM data.

To expedite the clinical translation of CAFs in GBM, we devised a novel nomogram that integrates both the risk profile and clinicopathological parameters. This integrated approach promises a more nuanced stratification of GBM patients and enhances the precision of prognostic assessments. Furthermore, we explored the immune landscape and immunotherapy responsiveness associated with CAFs‐related signatures, providing fresh insights and predictive tools that could inform GBM management strategies and treatment decisions.

## 2. Materials and Methods

### 2.1. Data Collection and Processing

Relevant datasets for this study were acquired from publicly accessible repositories, including the Gene Expression Omnibus (GEO), The Cancer Genome Atlas (TCGA), and the China Glioblastoma Genome Atlas (CGGA). Preprocessing of the scRNA‐seq data was carried out using the “Seurat” package (v4.0.2). The proportion of mitochondrial gene expression was calculated with the PercentageFeatureSet function. To assess potential technical artifacts, correlation analyses were performed to examine the associations between sequencing depth and both mitochondrial gene content and total cellular expression levels. Normalization of the data was conducted using the LogNormalize method.

### 2.2. Definition of CAFs

We re‐analyzed GBM scRNA‐seq data with the “Seurat” pipeline to systematically profile CAFs. Batch effects across the four samples were harmonized using the FindIntegrationAnchors function. Nonlinear dimensionality reduction was carried out with uniform manifold approximation and projection (Umap), based on 30 principal components and a cluster resolution set to 1. Cell subgroups were identified via the FindNeighbors and FindClusters functions (dims = 30, resolution = 0.2) [19]. Further visualization was achieved using t‐distributed stochastic neighbor embedding (t‐SNE) implemented in the RunTSNE function. Fibroblasts were defined based on expression of four established markers: ACTA2, PDGFRB, THY1, and COL1A1. These cells were subsequently re‐clustered applying the same FindNeighbors and FindClusters workflow. A separate t‐SNE projection was generated for fibroblast subpopulations. To identify cluster‐specific marker genes, we performed inter‐cluster comparisons with the FindAllMarkers tool, applying thresholds of |logFC| ≥ 0.5, min.pct ≥ 0.35, and adjusted *p*‐value < 0.05 [20]. Marker genes for each CAFs cluster were then subjected to Kyoto Encyclopedia of Genes and Genomes (KEGG) pathway enrichment analysis using the clusterProfiler package [21]. Additionally, inferential copy number variation (CNV) profiling was conducted with the CopyKAT (v1.0.8) tool to distinguish malignant from non‐malignant cells within each sample [22, 23].

### 2.3. Identification of CAFs Hub Genes

Differentially expressed genes (DEGs) associated with CAFs clusters were identified using the “limma” package, and their expression correlations were assessed [24]. To further pinpoint genes with prognostic significance, univariate Cox regression analysis was carried out with the survival package. The Least Absolute Shrinkage and Selection Operator (LASSO) regression technique was applied to refine the candidate gene set and mitigate overfitting. Subsequently, a multivariate Cox regression analysis employing a stepwise selection approach was utilized to develop a validated prognostic signature [25].

### 2.4. Development and Validation of the Risk Scoring Model

Based on the multivariate Cox regression results, a prognostic signature was established using the formula: risk score = ∑*βi* × Expi, where *i* represents each gene in the risk profile, expi indicates the expression level of gene *i*, and *βi* denotes the coefficient of gene *i* in the multifactor Cox model. To evaluate the predictive performance of this risk model, receiver operating characteristic (ROC) curves were generated and the area under the curve (AUC) was computed. Additionally, Kaplan–Meier survival analysis was conducted to compare outcomes between high‐ and low‐risk groups, confirming the value of the risk score as an independent prognostic factor [25].

### 2.5. Analysis of Tumor Immune Microenvironment

To characterize the tumor immune microenvironment in GBM, the “estimate” R package was applied to compute StromalScore, ImmuneScore, EstimateScore, and tumor purity for each sample. The resulting scores were visualized using the “ggpubr” package. Immune cell infiltration profiles across TCGA tumor samples were derived from the TIMER2.0 platform, incorporating multiple deconvolution algorithms—including TIMER, CIBERSORT, EPIC, and MCPCOUNTER—to estimate abundances of various cell types [26]. Differences in immune cell composition between high‐ and low‐risk patient groups were depicted graphically using the “pheatmap” package.

### 2.6. Risk Signature and Nomogram Development

To develop a clinically applicable predictive tool, univariate and multivariate Cox regression analyses were performed incorporating both clinicopathological variables and the risk signature. Variables that demonstrated statistical significance (*p*  < 0.05) in the multivariate analysis were selected to construct a nomogram for predicting GBM patient outcomes using the “rms” package in R [27]. The predictive accuracy of the nomogram was evaluated using calibration curves, while its clinical utility was assessed via decision curve analysis (DCA).

### 2.7. Analysis of Gene Mutations and Gene Set Enrichment

Enrichment scores for HALLMARK signaling pathways and KEGG pathways were compared between high‐ and low‐risk groups using the “GSVA” R package (1.42.0). Potential disparities in pathway activity were assessed to uncover biological differences underlying risk stratification. Furthermore, single nucleotide variant (SNV) profiles and CNV patterns of genes included in the risk model were systematically examined.

### 2.8. Assessment of Response to Immunotherapy

Transcriptomic data from the IMvigor210 cohort were combined with clinical information from GBM patients treated with the anti‐PD‐1/PD‐L1 agent atezolizumab. Furthermore, the GSE78220 dataset, which contains pre‐treatment transcriptomic profiles from melanoma patients receiving anti‐PD‐1/PD‐L1 immune checkpoint blockade therapy, was incorporated into the analysis to evaluate predictive biomarkers of immunotherapy response [28].

### 2.9. Drug Sensitivity Analysis

Chemotherapeutic drug sensitivity was predicted for each tumor sample using the “pRRophetic” R package, which leverages data from the cancer drug sensitivity genomics database (GDSC) [25]. Half‐maximal inhibitory concentration (IC_50_) values for specific chemotherapeutic agents were estimated using ridge regression. The predictive performance of the model was assessed through 10‐fold cross‐validation based on the GDSC training set. Default parameters were employed throughout the analysis, including the “combat” adjustment for batch effects, and repeated gene expression measurements were averaged to ensure robustness.

### 2.10. Statistical Analysis

All statistical analyses were conducted using R (version 4.2.3). Independent prognostic factors for overall survival were identified through univariate and multivariate Cox regression analyses. The prognostic model’s predictive accuracy was evaluated by ROC curve analysis. Wilcoxon test was used to compare tumor‐infiltrating immune cell levels. Visualizations were generated with the “ggplot2”, “pheatmap”, and “forestplot” packages. Statistical significance was defined as *p*  < 0.05.

## 3. Results

### 3.1. Clustering and Identification of CAFs in scRNA‐Seq Samples

To fully comprehend the different types and functions of CAFs within the glioma TME, we downloaded a gene expression dataset (GSE162631) comprising isolated cells from tumor centers and surrounding tissues of four GBM patients. After initial screening, we obtained a total of 69,145 cells from the scRNA‐seq data, the counts in each cell ranging from 100 to 7500. Following preprocessing, we identified 57,813 cells, having excluded those with over 30% mitochondrial content. We focused on 50 of the most distinct components for further exploration. After applying logarithmic normalization and dimensionality reduction, we identified 30 subpopulations (Figure 1A).

Figure 1CAFs clusters were identified based on scRNA‐seq data from GBM patients. (A) The GEO microarray (GSE162631) obtained from the GEO database, after a series of screening and clustering, the topography of cell distribution and fibroblastogene‐based marker gene expression of the four samples. (B) Umap distribution of the five newly discovered fibroblast clusters after further exclusion and clustering. (C) Bubble plot of the five most expressed marker genes in the five fibroblast clusters. (D) The expression, number and proportion of five fibroblast clusters in tumor and adjacent tissues. (E) KEGG pathway enrichment analysis of five fibroblast subsets. (F) The simulation package predicts the Umap distribution of malignant and non‐malignant cells.

**Figure:**
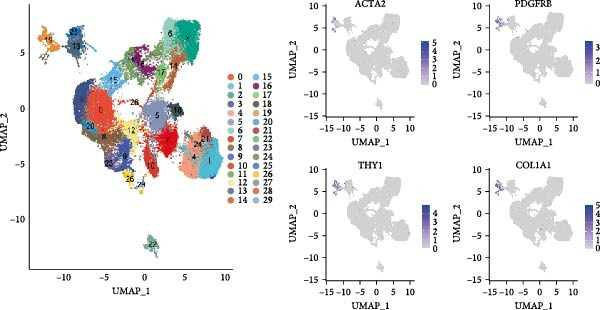
(A)

**Figure:**
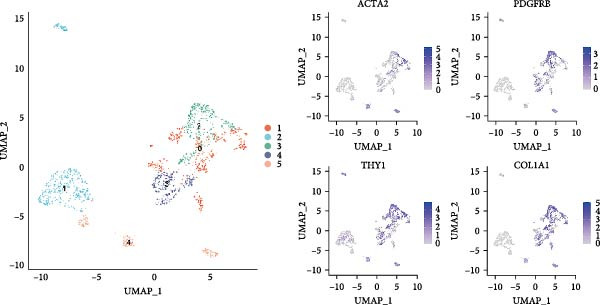
(B)

**Figure:**
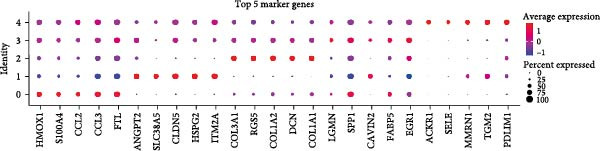
(C)

**Figure:**
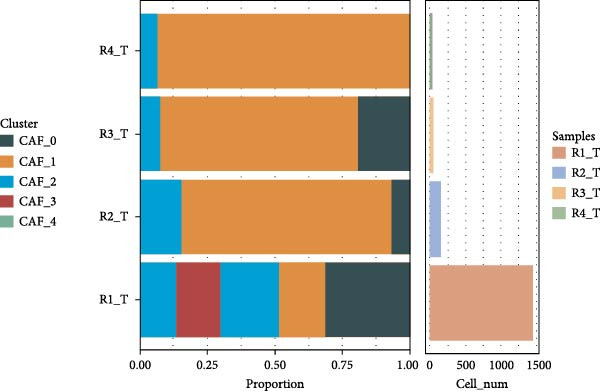
(D)

**Figure:**
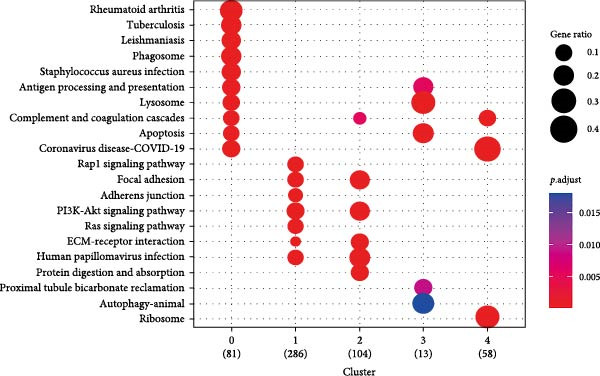
(E)

**Figure:**
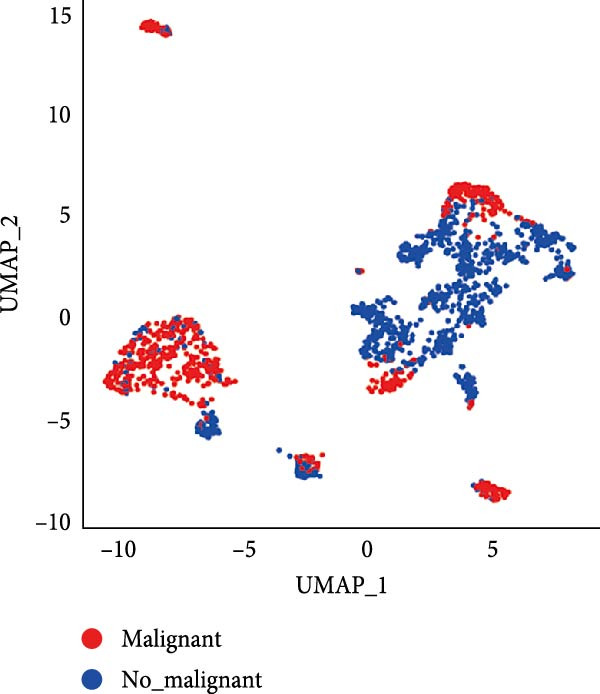
(F)

To obtain more precise CAFs clusters with stronger associations to gliomas, we selected three clusters characterized by high expression of cancer‐related marker genes: actin alpha 2 (ACTA2), platelet‐derived growth factor receptor beta (PDGFRB), THY1, and collagen type I alpha 1 chain (COL1A1), specifically clusters 19, 25, and 27. Cells from these three CAFs subpopulations were then separated for additional downstream analyses, which included further clustering and dimensionality reduction. Employing an identical algorithmic approach to clustering, five distinct CAFs clusters were discerned (CAF_0, CAF_1, CAF_2, CAF_3, and CAF_4), as illustrated in Figure 1B. To understand the functions and signatures of each CAFs cluster, we identified 1069 differentially expressed genes (DEGs) across the five clusters. The top five DEGs with the highest expression in each cluster were considered marker genes, and their expression levels are displayed in Figure 1C.

We then analyzed the distribution of these five clusters across each sample. RT_1 exhibited the largest number of CAFs, with CAF_3 and CAF_4 being exclusive to RT_1, while CAF_0, CAF_1, and CAF_2 were present in all samples (Figure 1D). Additionally, pathway enrichment analysis using KEGG indicated that the DEGs were significantly associated with multiple biological pathways. Specifically, the DEGs associated with CAF_0 were linked to rheumatoid arthritis, tuberculosis, and leishmaniasis, while DEGs from CAF_1, CAF_2, CAF_3, and CAF_4 present in tumor samples were associated with apoptosis, focal adhesion, adherens junctions, the Phosphatidylinositol‐3 kinase−protein kinase B (PI3K−Akt) signaling pathway, protein digestion and absorption, the Ras signaling pathway, Extracellular Matrix–receptor interaction, and autophagy. This indicates that the enrichment and function of CAFs may be altered in the TME of GBM (Figure 1E). Based on CNV characteristics, the five CAFs clusters were composed of 1533 tumor cells and normal cells, with CAF_1 primarily comprising malignant tumor cells, while the other clusters showed no significant differences between benign and malignant cells (Figure 1F).

### 3.2. Expression of Relevant Pathways in CAFs and Their Association With Prognosis

To validate the accuracy of our classification of CAFs clusters and their correlation with glioma, considering that the TME may influence the morphology and function of CAFs during GBM progression, we compared the enrichment of tumor‐related signaling pathways among CAFs in malignant versus non‐malignant tumors. Gene Set Variation Analysis (GSVA) analysis of 10 tumor‐related pathways across different CAFs clusters revealed that CAF_1 contained the highest number of malignant cells, with significantly elevated GSVA scores in malignant cells compared to non‐malignant cells across all CAFs clusters. Notably, CAF_1 clusters exhibited high expression levels across most pathways, regardless of the malignant cellular context (Figure 2A). This finding aligns with our CNV predictions, highlighting a significantly higher proportion of malignant cells in CAF_1 compared to other clusters, with distinct differences observed among CAF_0, CAF_2, CAF_3, and CAF_4 (Figure 2B).

Figure 2Characteristics of tumor‐associated pathways in CAFs clusters. (A) Heat map of the top 10 tumor‐associated pathways most enriched in the five CAFs cell clusters. (B) Comparison between clusters based on the proportion of malignant and non‐malignant cells. (C–G) Comparison of pathways between malignant and non‐malignant cells based on GSVA score in CAF_0, CAF_1, CAF_2, CAF_3, and CAF_4.

**Figure:**
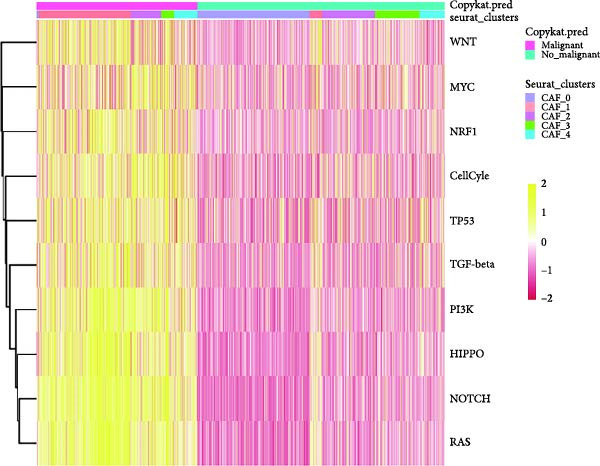
(A)

**Figure:**
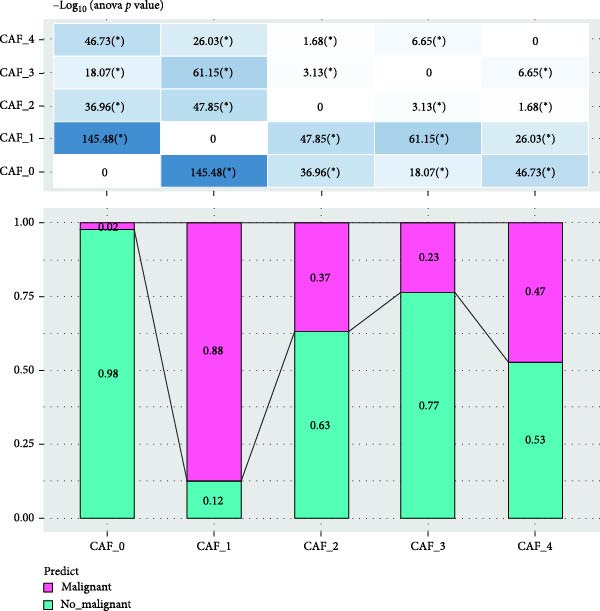
(B)

**Figure:**
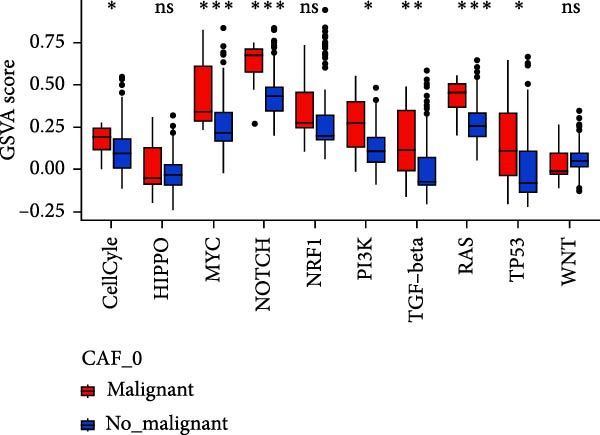
(C)

**Figure:**
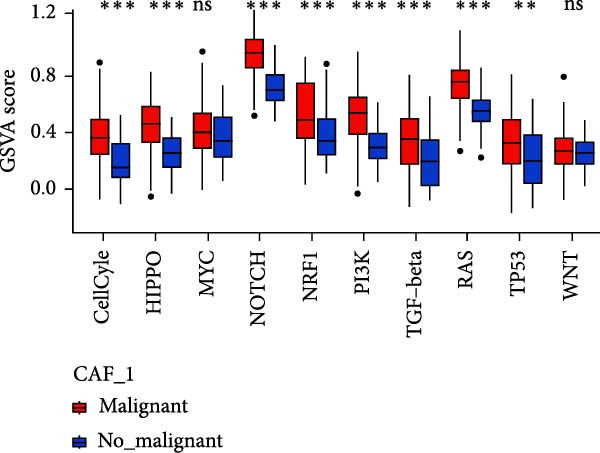
(D)

**Figure:**
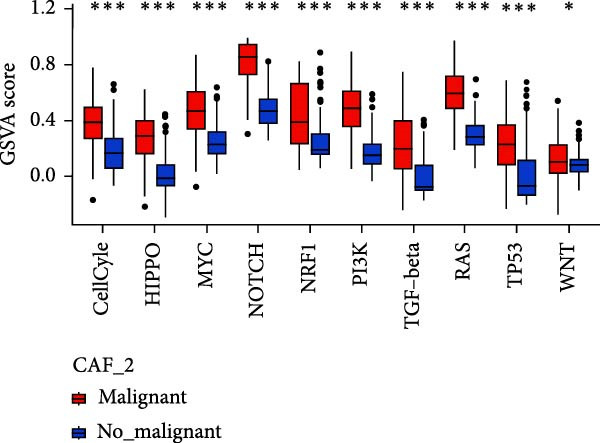
(E)

**Figure:**
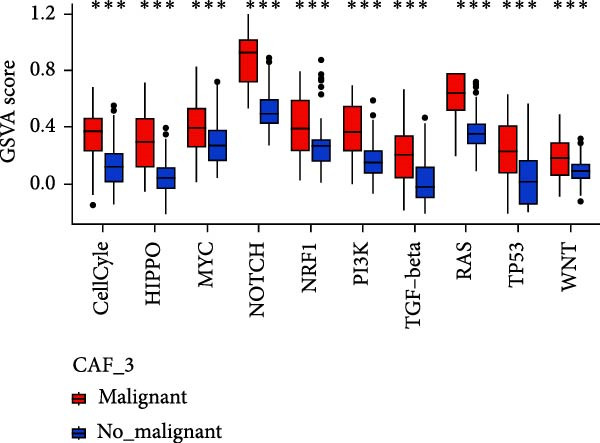
(F)

**Figure:**
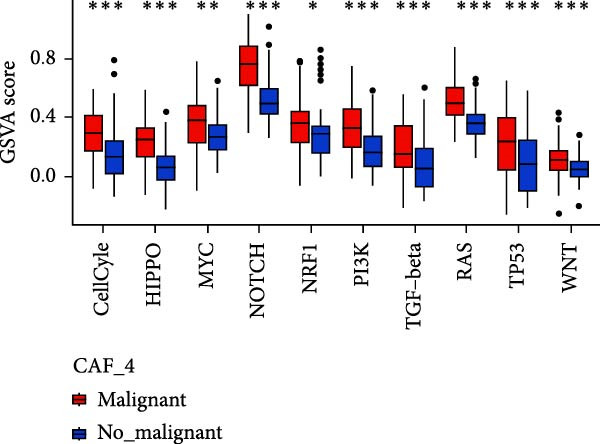
(G)

Subsequently, GSVA scores for the 10 pathways were separately calculated for malignant and non‐malignant cells within each CAFs cluster (Figure 2C–G), showing that tumor samples scored significantly higher than normal samples in most pathways across all clusters. After distinguishing between malignant and non‐malignant cells, we utilized ssGSEA to calculate HALLMARK enrichment scores for individual cells in the samples. The results indicated that most pathways were enriched in malignant cells. The previous experimental results showed that the proportion of malignant cells in RT‐1 was not high, and the malignant degree of the samples was low, so the score result of RT‐1 may be abnormal. After excluding RT_1, the enrichment of most pathways was significantly different between benign and malignant cells (Supporting Information: Figure S1A).

To assess the relationship between CAFs clustering and prognosis, we calculated ssGSEA scores for each CAFs cluster marker gene using data from the TCGA cohort (Supporting Information: Figure S1D). In every CAFs cluster, tumor samples scored significantly higher than normal samples, as demonstrated by the results. Utilizing the survminer R software package, we categorized GBM samples in the TCGA cohort into low and high CAFs score groups based on optimal cutoffs (Supporting Information: Figure S1C). Patients in the low CAFs score group experienced significantly worse prognoses, implying that CAFs may serve as a valuable indicator of GBM progression.

### 3.3. Identification of Key CAFs Genes and Construction of Risk Score Model

As previously mentioned, we have confirmed the significant role of CAFs in the TME of GBM. We aimed to construct a risk characteristic model to predict treatment responses and prognosis for GBM. Utilizing samples from the GEO database (GSE162631), by analyzing normal and tumor tissues, we identified 4556 DEGs, including 2484 that were upregulated and 2070 that were downregulated (Figure 3A). Among these, 407 genes exhibited significant associations with prognostic CAFs clusters, indicating their potential as targets for CAFs’ actions within the GBM microenvironment.

Figure 3The sample (GSE162631) downloaded from the GEO database was used to obtain new risk signature structures of multiple CAFs‐related genes. (A) Volcano plot of DEGs between tumor and normal samples in TCGA cohort. (B) Volcano plot of prognostic genes obtained by univariate Cox regression analysis. (C) Trajectory and distribution of each independent variable. Each gene in the risk signature has associated multivariate Cox coefficients. (D) Cox coefficient analysis of six hub genes. According to the Cox coefficient and risk equation, the benign and malignant status of each CAFs gene was estimated. (E) Survival curves and risk attributes of patients in the TCGA cohort are represented by K‐M and ROC curves. (F) Survival curves and risk attributes of patients in the CGGA cohort are represented by K‐M and ROC curves. (G) Survival curves and risk attributes of patients in the GSE83300 cohort are represented by K‐M and ROC curves.

**Figure:**
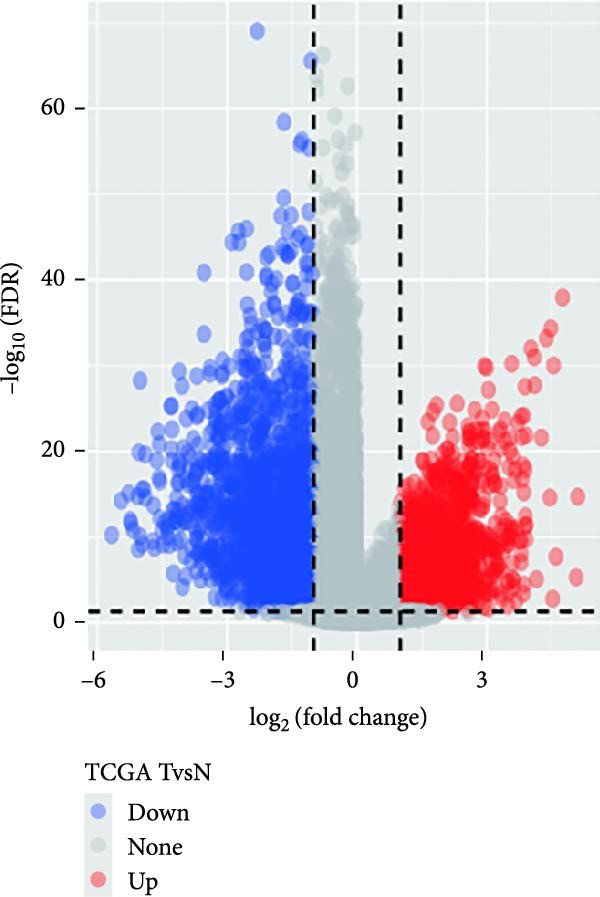
(A)

**Figure:**
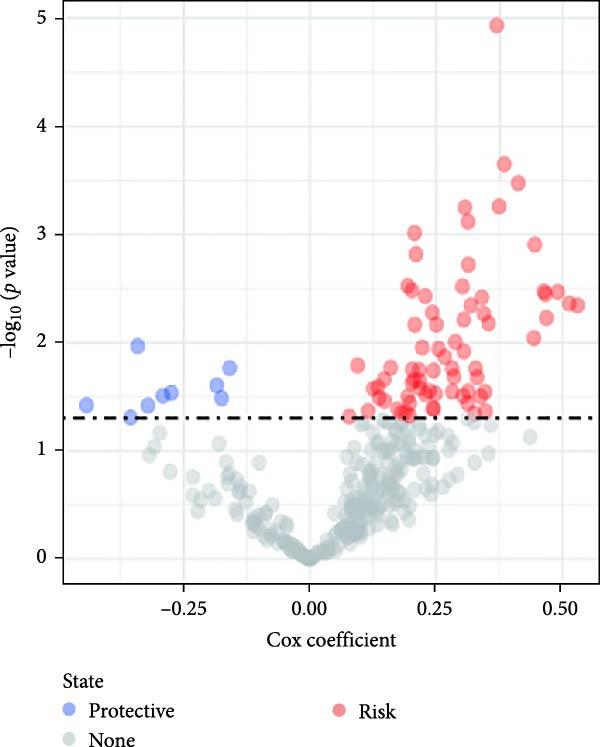
(B)

**Figure:**
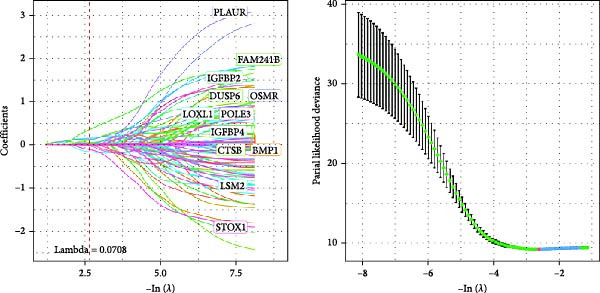
(C)

**Figure:**
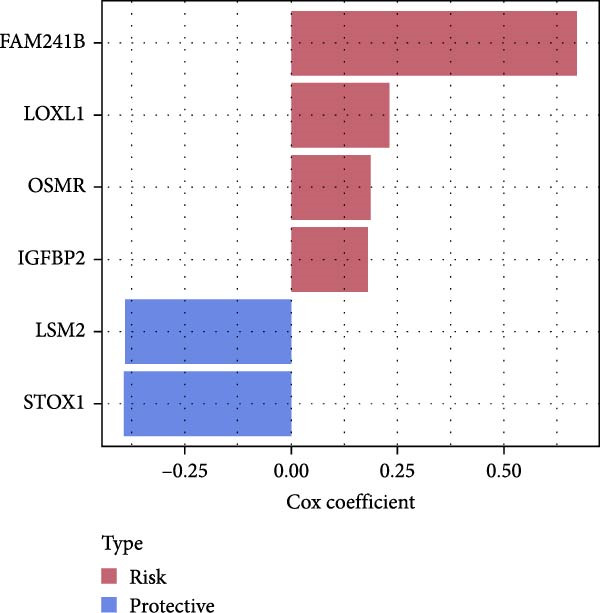
(D)

**Figure:**
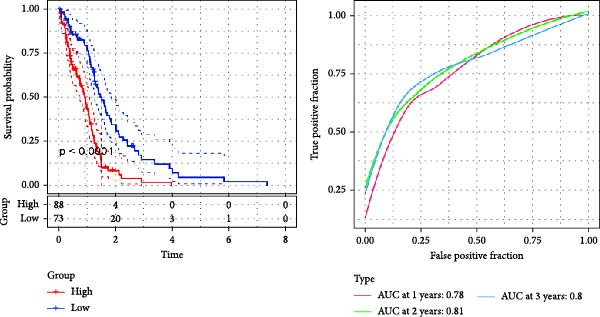
(E)

**Figure:**
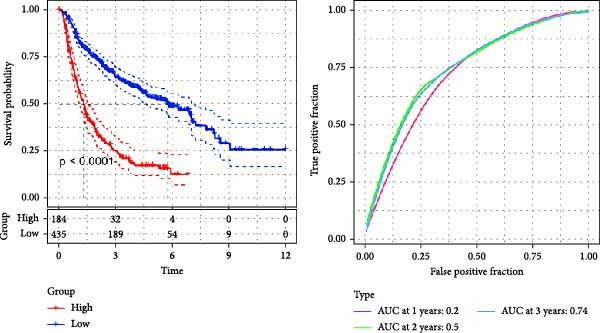
(F)

**Figure:**
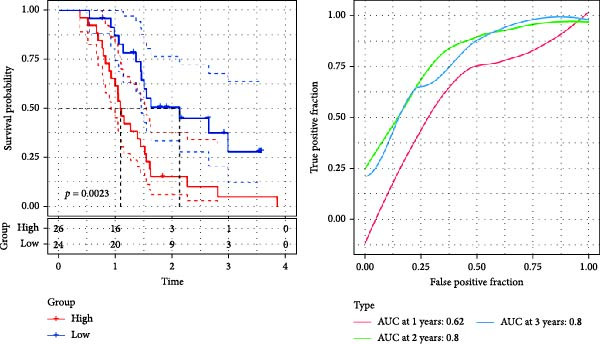
(G)

Through analyses of Gene Ontology (GO) and KEGG enrichment, we found that these genes were concentrated in pathways related to focal adhesion, extracellular matrix structural constituents, collagen‐containing extracellular matrices, and external encapsulating structure organization, among others (Supporting Information: Figure S1B). Further evaluation using univariate Cox regression analysis revealed 83 genes with notable prognostic value (Figure 3B). We used Lasso Cox regression analysis with a *λ* value of 0.0708 to narrow down the list of candidate genes, resulting in a selection of 6 key genes. Subsequently, we constructed a risk profile using stepwise regression via multiple Cox regression analysis (Figure 3C). This signature comprises the following 6 hub genes: Family with Sequence Similarity 241 Member B (FAM241B), LSM2 Homolog (LSM2), Insulin‐Like Growth Factor Binding Protein 2 (IGFBP2), Lysyl Oxidase Like 1 (LOXL1), Oncostatin M Receptor (OSMR), and Storkhead Box 1 (STOX1). The conclusive risk calculation is given by: RiskScore = 0.672 × FAM241B − 0.391 × LSM2 + 0.180 × IGFBP2 + 0.232 × LOXL1 + 0.188 × OSMR − 0.393 × STOX1. Notably, LSM2 and STOX1 are considered protective factors, with higher expression correlating with better prognosis, while increased expression of the other genes correlates with higher risk scores (Figure 3D). Risk scores for each sample were determined post Z‐score normalization, allowing us to categorize patients into high‐risk and low‐risk groups. Analysis using Kaplan–Meier survival and AUC methods revealed that patients at low risk had considerably better survival outcomes than those at high risk (Figure 3E).

To validate the generalizability of this model, we respectively introduced the GSE74187 (60 cases of GBM) and GSE83300 (50 cases of GBM) datasets from the GEO database, as well as the dataset containing 6883 GBM samples from the CGGA database as validation queues. Results from the TCGA cohort and the three validation datasets indicated that the risk scores can effectively distinguish between patients at high risk and those at low risk, and individuals with low risk consistently demonstrated superior survival compared to high‐risk patients, highlighting the robust predictive capability of our RiskScore‐based model. Furthermore, AUC values for the 1‐ to 3‐year survival model in the TCGA cohort were found to be between 0.78 and 0.80, while AUC values ranged from 0.72 to 0.74 in CGGA, 0.65 to 0.88 in GSE74187, and 0.62 to 0.80 in GSE83300, indicating strong predictive performance (Figure 3F,G, Supporting Information: Figure S1E).

### 3.4. Correlation Analysis Between Risk Score and Prognosis and Construction of Nomogram

To enhance the translational potential and prognostic accuracy of the CAFs‐based risk model, we integrated the risk score with clinical pathological features (such as age and gender) and conducted univariate and multivariate Cox regression analyses. Univariate analysis identified risk scores and age as significant independent prognostic factors in the GBM risk model (Figure 4A). Multivariate analysis further confirmed that risk characteristics were the most critical independent prognostic factors for GBM (*p*  < 0.001) (Figure 4B). Previous studies have indicated that GBM incidence is influenced by age, gender, and race, peaking in individuals aged 75–84, with a higher prevalence in men compared to women, and the highest rates observed in non‐Hispanic whites [5]. After incorporating multiple factors, we designed an innovative nomogram that factors in stage and risk scores. Calibration curves showed good agreement between the predicted survival probabilities and actual observed outcomes, indicating high predictive accuracy of the model. DCA revealed that the nomogram provided significantly greater clinical net benefit in identifying high‐risk patients compared to using the risk score alone or traditional staging methods (Figure 4C). Time‐dependent ROC analysis across each validation cohort revealed AUC values approaching 0.8 for both the nomogram and risk score, indicating high accuracy and predictive performance (Figure 4D).

Figure 4A new nomogram incorporating risk characteristics was developed. (A–B) Outcomes of Cox regression analysis, both univariate and multivariate, based on risk score, age, sex, and race. (C) Construct a nomogram by combining risk score, age, sex, and race. The nomogram was calibrated and validated using patient survival data at 1 and 2 years. (D) The predictive power of the nomogram, risk score, age, sex, and race were evaluated by time‐ROC analysis.

**Figure:**
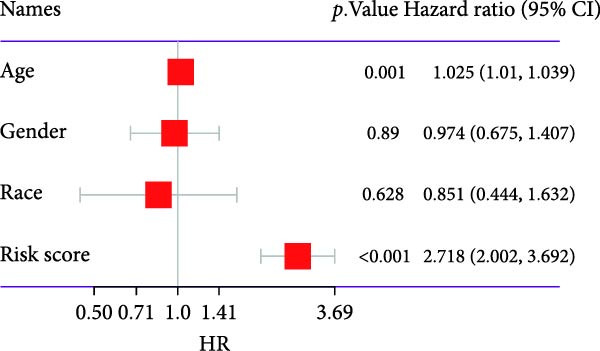
(A)

**Figure:**
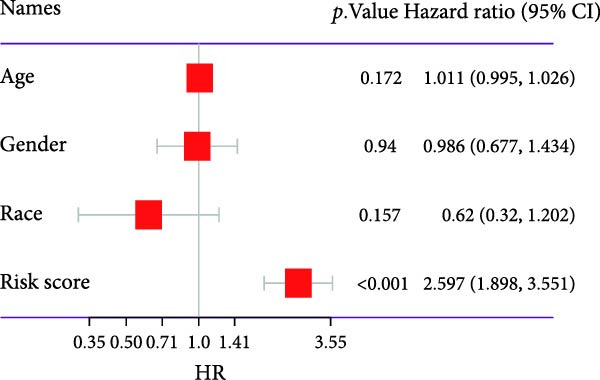
(B)

**Figure:**
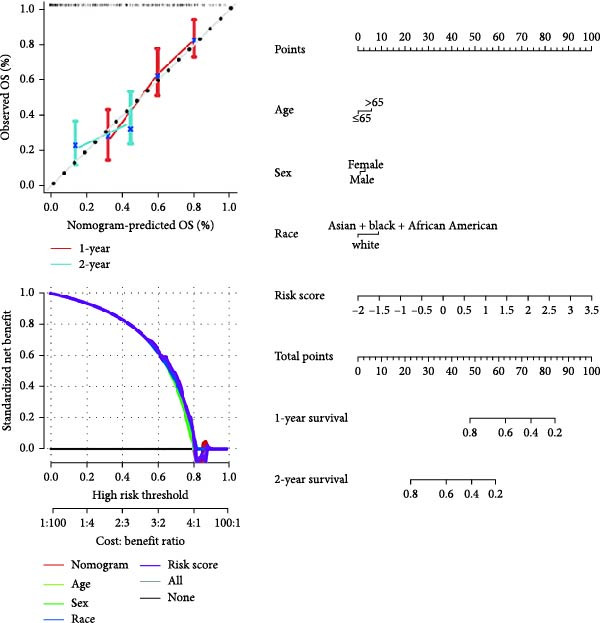
(C)

**Figure:**
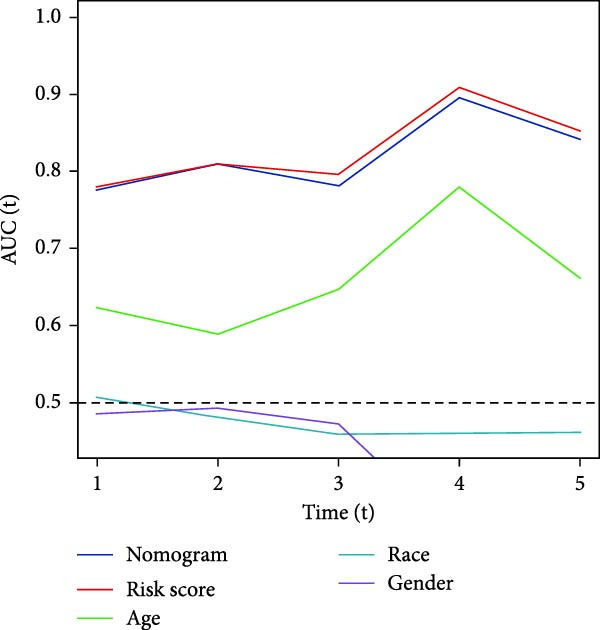
(D)

### 3.5. Mutation and Pathway Analysis of Key Genes

To explore in more detail the potential molecular mechanisms of genes linked to CAFs in GBM, we investigated SNV and CNV mutations of several key genes in glioma using the TCGA cohort. A limited number of SNV mutations were identified in OSMR, STOX1, and LOXL1, while no SNV mutations were detected in FAM241B, LSM2, or IGFBP2 (Figure 5A). Further pathway enrichment analysis showed that these key genes were broadly involved in classic signaling pathways, including tumor protein 53 (TP53), RTK‐RAS, and PI3K (Figure 5B). In terms of CNV, only a small number of samples showed copy number gains or losses, primarily in OSMR (Figure 5C). We then evaluated the co‐occurrence of mutations between the six CAFs‐related key genes and the top 10 most frequently mutated genes in GBM. As shown in Figure 5D, there was no significant co‐mutation trend, suggesting these genes might function independently to promote tumor progression, potentially through mutually exclusive mechanisms. Moreover, correlation analysis among these key genes indicated a certain degree of interrelation (Figure 5E), implying they may form a synergistic regulatory network that collectively mediates GBM microenvironment remodeling and tumor progression.

Figure 5(A) SNV variants in six hub genes. (B) Pathways affected by gene mutations. (C) CNV variants in six hub genes. (D) Co‐occurrence probability of key genes with SNV mutations and the 10 most mutated genes. (E) Correlation between hub genes.

**Figure:**
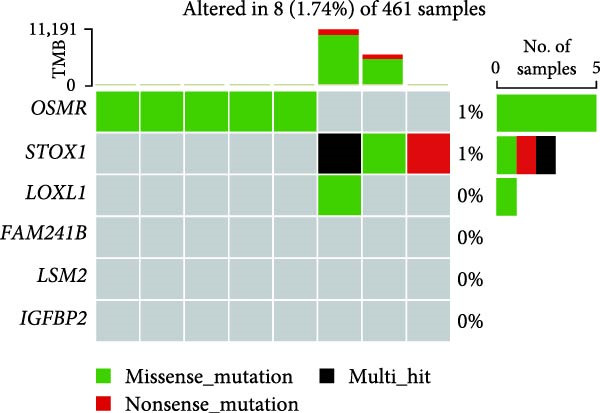
(A)

**Figure:**
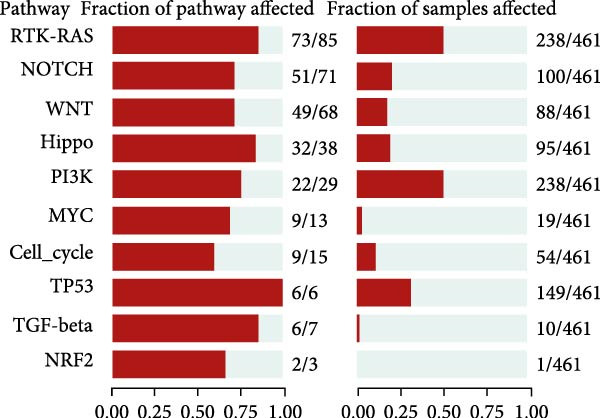
(B)

**Figure:**
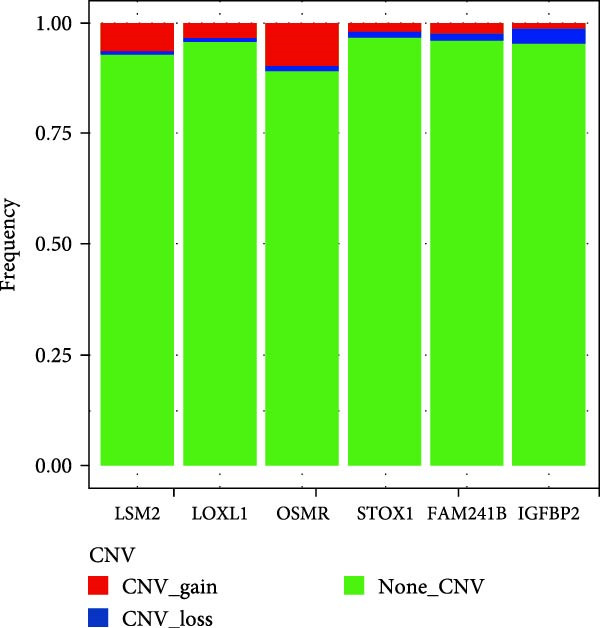
(C)

**Figure:**
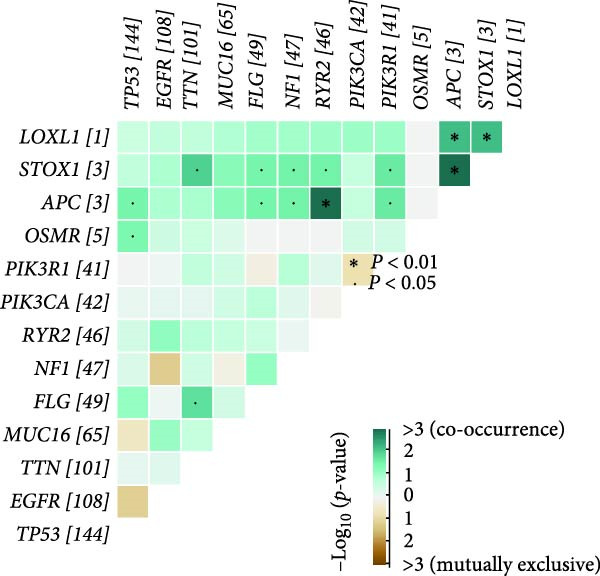
(D)

**Figure:**
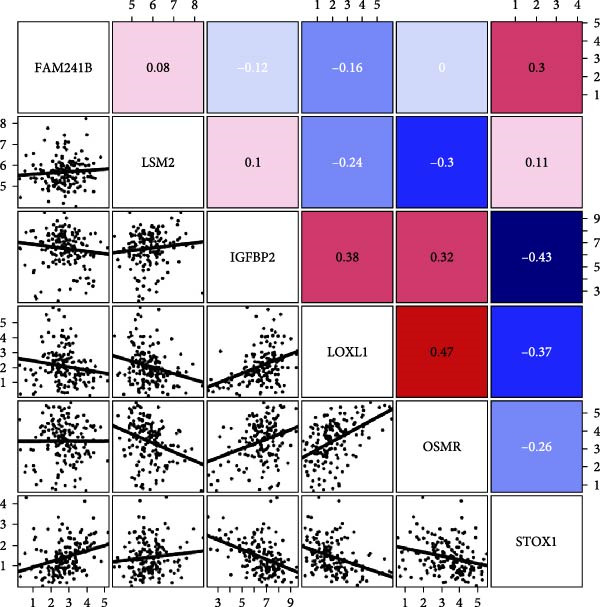
(E)

Additionally, we utilized KEGG and HALLMARK analyses to investigate potential pathways associated with each risk gene. Our findings revealed that four pathways—UV_RESPONSE_DN, epithelial‐mesenchymal transition (EMT), APOPTOSIS, and TNFA_SIGNALING_VIA_NFKB—were significantly correlated with the six hub genes. Among them, OSMR, LOXL1 and IGFBP2 were highly expressed across multiple pathways. Notably, FAM241B and LSM2 exhibited negative correlations with certain pathways, suggesting that they may play inhibitory or regulatory roles under specific conditions by modulating opposing signals (Figure 6A,B). In the KEGG pathway enrichment analysis, 14 pathways including ADHERENS_JUNCTION and FOCAL_ADHESION showed significant associations with the six key genes. Similarly, OSMR, LOXL1, and IGFBP2 were positively correlated with multiple pathways such as FOCAL_ADHESION and GLYCOSAMINOGLYCAN_BIOSYNTHESIS_KERATAN_SULFATE, whereas LSM2 and FAM241B demonstrated negative correlations (Figure 6C,D). These findings indicate that OSMR, LOXL1, and IGFBP2 likely play crucial roles in regulating the TME, while FAM241B and LSM2 may exert antagonistic functions under certain conditions, reflecting the potential functional heterogeneity of CAFs.

Figure 6Gene set enrichment analysis. (A–D) Correlation analysis of six genes and related pathways in KEGG and HALLMARKER databases.

**Figure:**
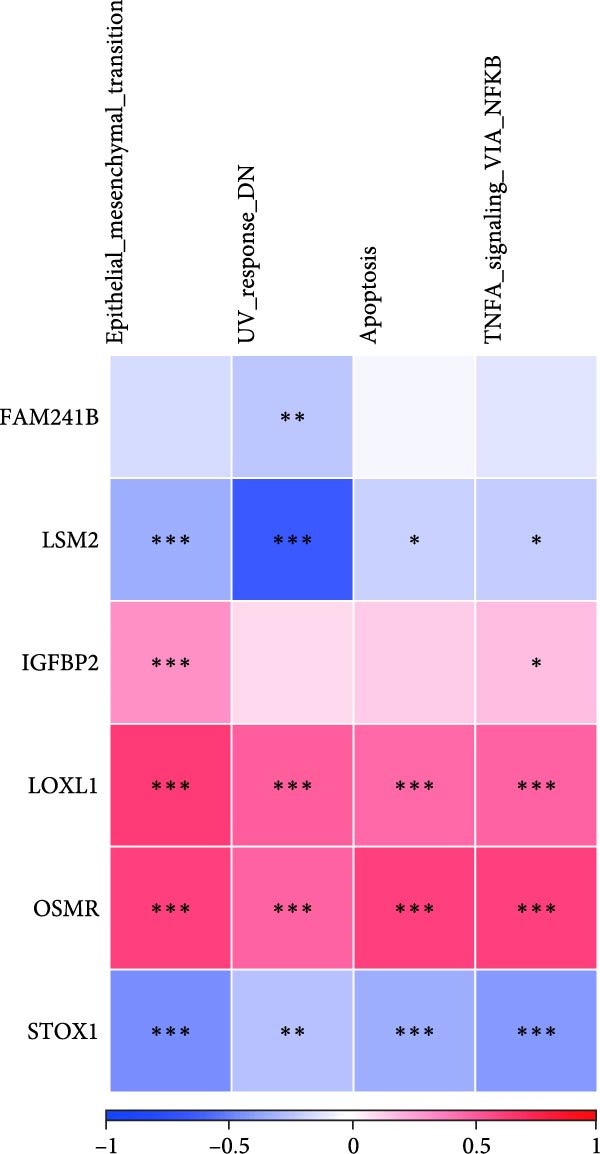
(A)

**Figure:**
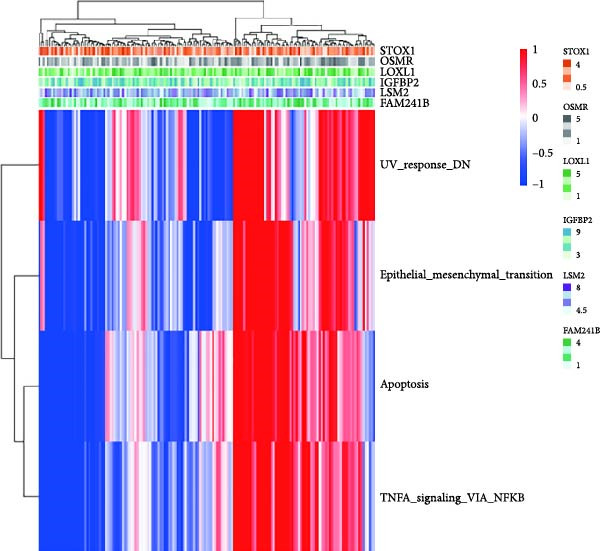
(B)

**Figure:**
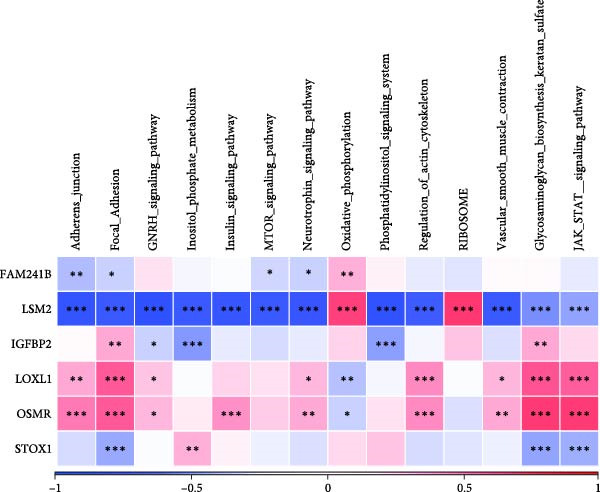
(C)

**Figure:**
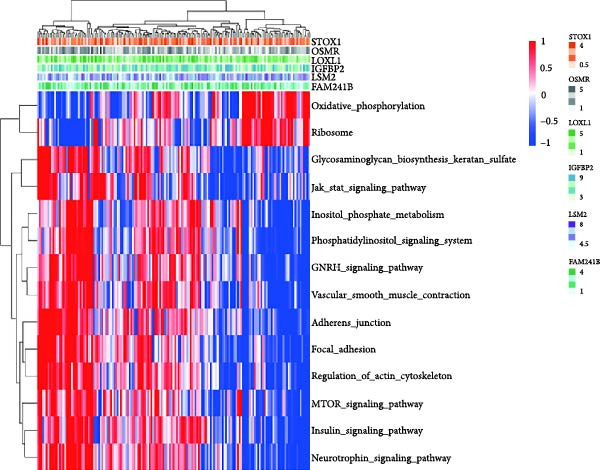
(D)

### 3.6. Immune Infiltration Status and the Association of Risk Genes With Immune Function

Next, we examined the potential functions of key genes in GBM, specifically their relationship with immunity. The data suggested that there was a significant positive relationship between LOXL1, OSMR, and immune scores (Figure 7A). We employed four immune infiltration methodologies—TIMER, CIBERSORT, EPIC, and MCPCOUNTER—to analyze immune dynamics in GBM and observed differences in immune cell expression between high‐risk and low‐risk groups [29] (Figure 7B). Upon isolating cells based on risk score gene expression levels, we found that cells with higher LSM2 expression had lower immune scores, while those with elevated OSMR expression had higher immune scores, reinforcing the roles of LSM2 and OSMR in glioma immunity (Figure 7C–H).

Figure 7Association analysis of key genes of CAFs and the immune microenvironment. (A) Correlation analysis between the expression levels of key genes in CAFs and ImmuneScore. (B) Use the TIMER, CIBERSORT, EPIC, and MCPCOUNTER algorithms to assess the variations in immune cell infiltration between groups with high and low risk scores. (C–H) Comparison of immune Core among the high and low expression groups of key CAFs genes.

**Figure:**
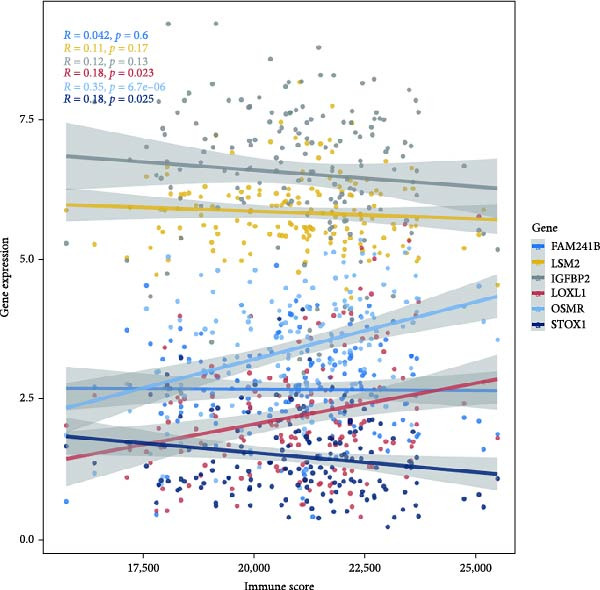
(A)

**Figure:**
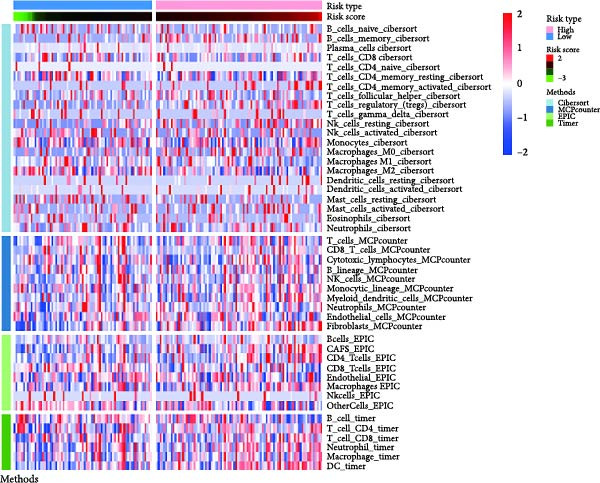
(B)

**Figure:**
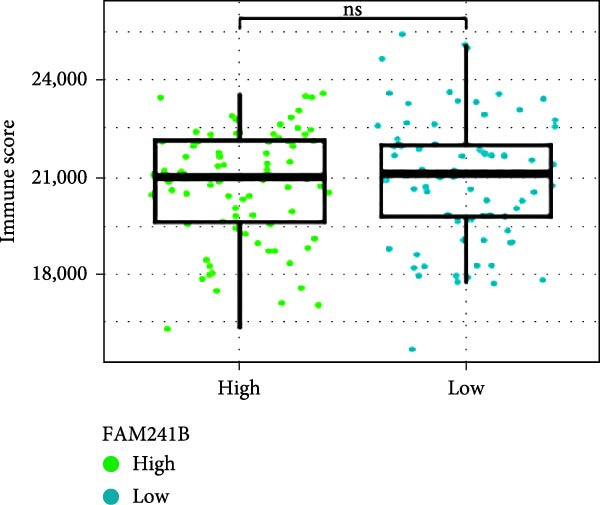
(C)

**Figure:**
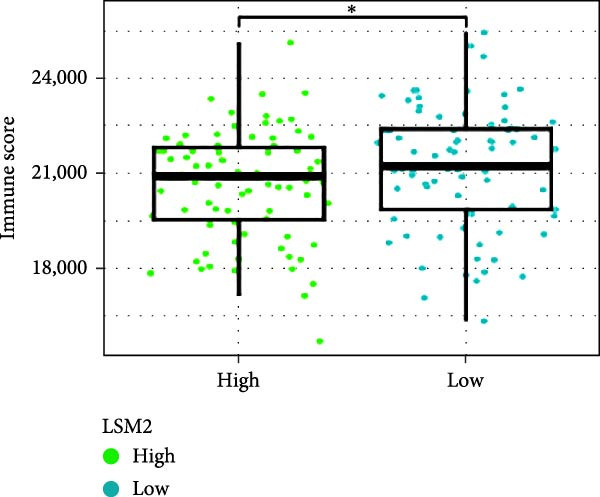
(D)

**Figure:**
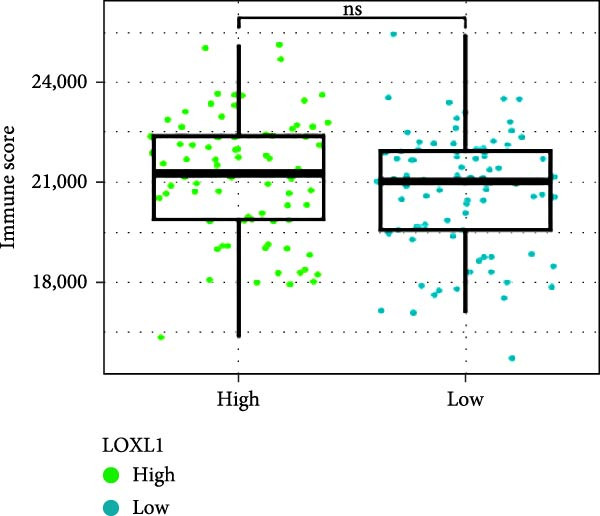
(E)

**Figure:**
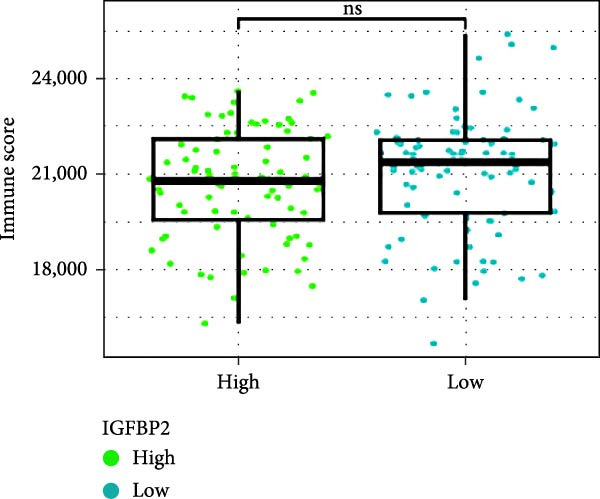
(F)

**Figure:**
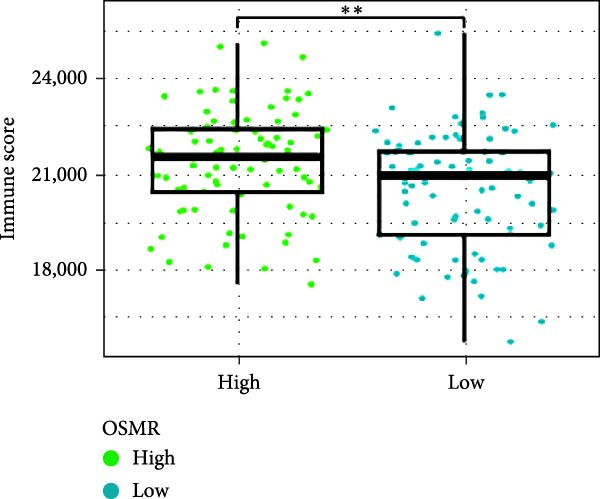
(G)

**Figure:**
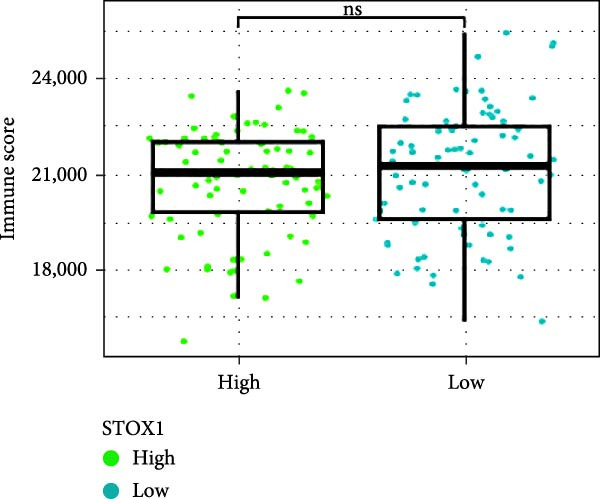
(H)

### 3.7. Risk Score Predicts Response to Immunotherapy and Drug Treatment

As a representative of tumor immunotherapy, PD‐1/PD‐L1 antibody drugs enhance T cell‐mediated killing of cancer cells and have demonstrated significant efficacy in various advanced malignancies [30, 31]. To assess the prognostic prediction capability of the CAFs‐related risk signature in individuals with GBM and its potential guidance for drug sensitivity and immunotherapy response, we conducted multidimensional validation of the model. Two cohorts receiving PD‐1/PD‐L1 blockade therapy, IMvigor210 and GSE78220, were selected for validation. Survival analysis showed that high‐risk patients did not benefit from immune checkpoint inhibition, whereas those in the low‐risk group experienced much better clinical results (Figure 8A). In the IMvigor210 cohort of 348 patients, we noted different reactions to anti‐PD‐1/PD‐L1 receptor blockers, encompassing stable disease (SD), partial response (PR), complete response (CR), and disease progression (PD). Notably, those with CR/PR had reduced risk scores relative to patients with SD/PD (Figure 8B). Furthermore, the low‐risk group had a smaller proportion of SD/PD patients compared to the high‐risk group (Figure 8C). Thus, our risk model serves as an excellent predictor for the effectiveness of PD‐1/PD‐L1 immunotherapy in GBM.

Figure 8Predictive value of the risk score for responsiveness to PD‐1/PD‐L1 blockade immunotherapy in the IMvigor210 and GSE78220 cohorts. (A) Prognostic differences between risk score groups in the IMvigor210 cohort. (B) Prognostic differences between risk score groups in the GSE78220 cohort. (C) Distribution of immunotherapy responses among different risk score groups in the IMvigor210 cohort. (D–I) Predictive outcomes of the risk model regarding sensitivity to anti‐tumor agents.

**Figure:**
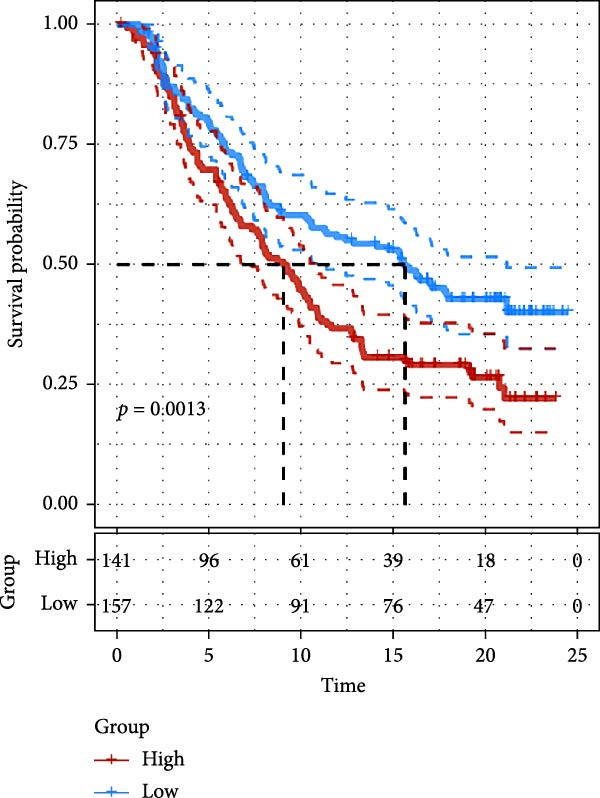
(A)

**Figure:**
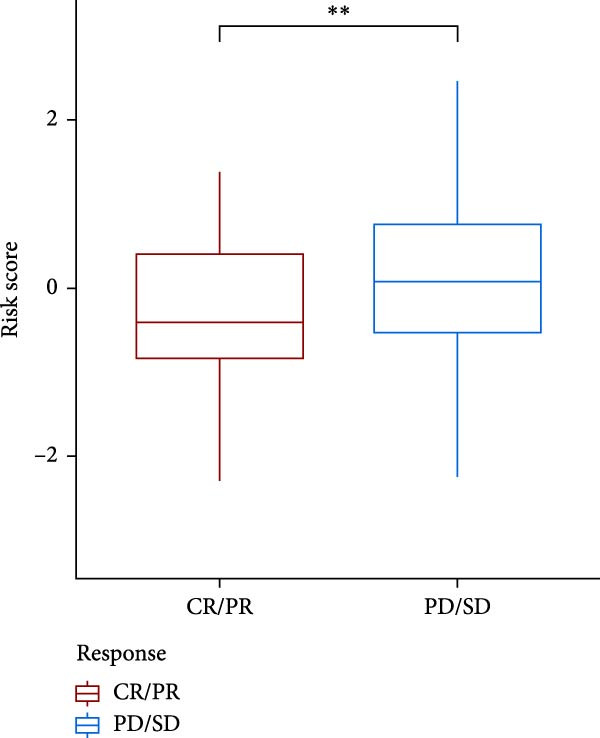
(B)

**Figure:**
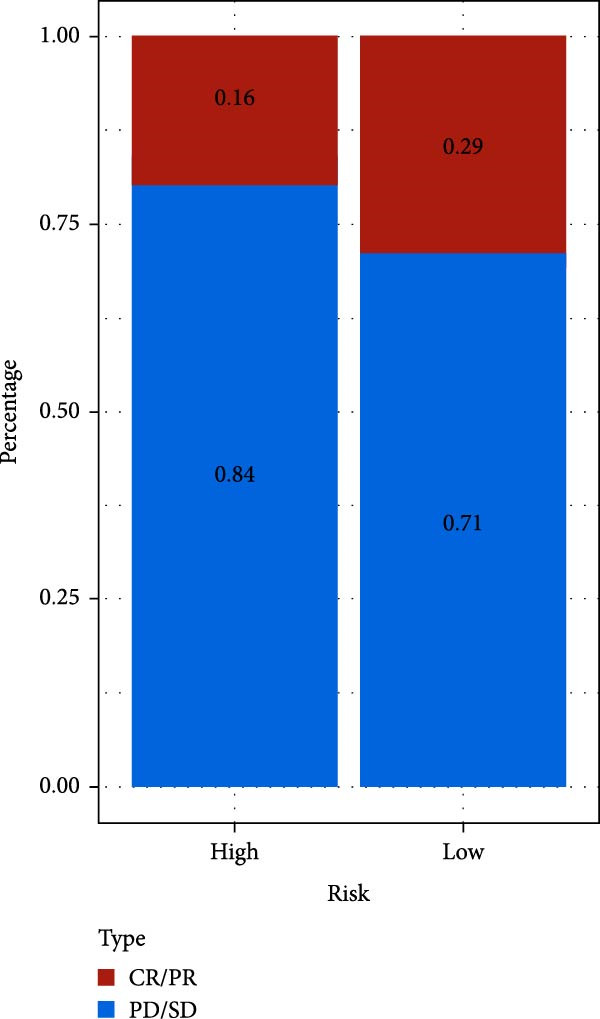
(C)

**Figure:**
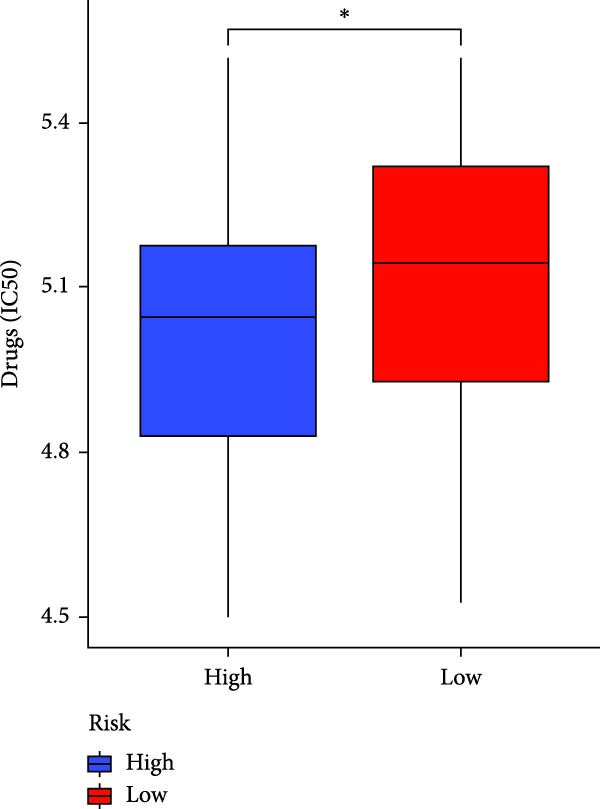
(D)

**Figure:**
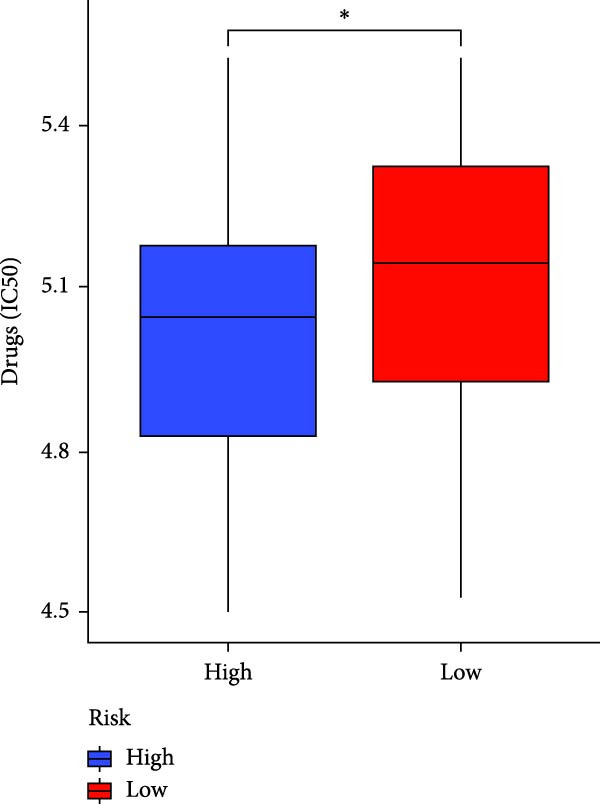
(E)

**Figure:**
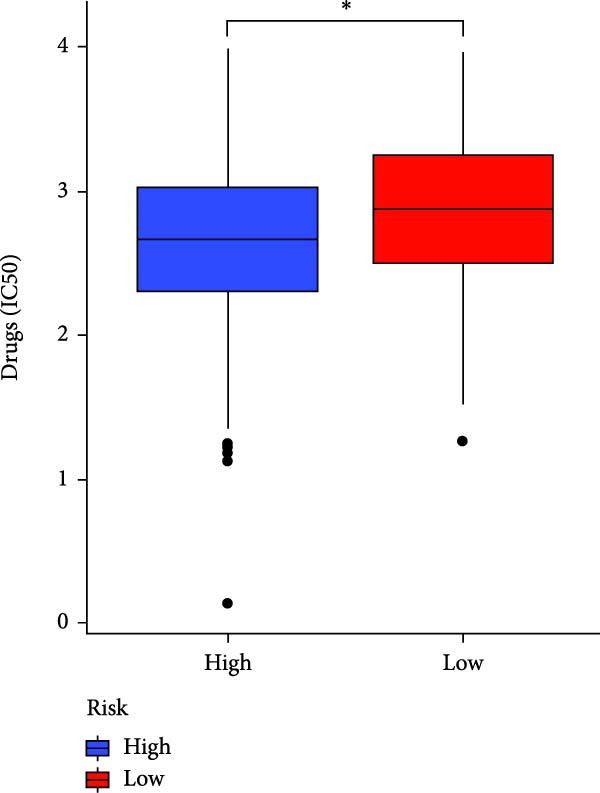
(F)

**Figure:**
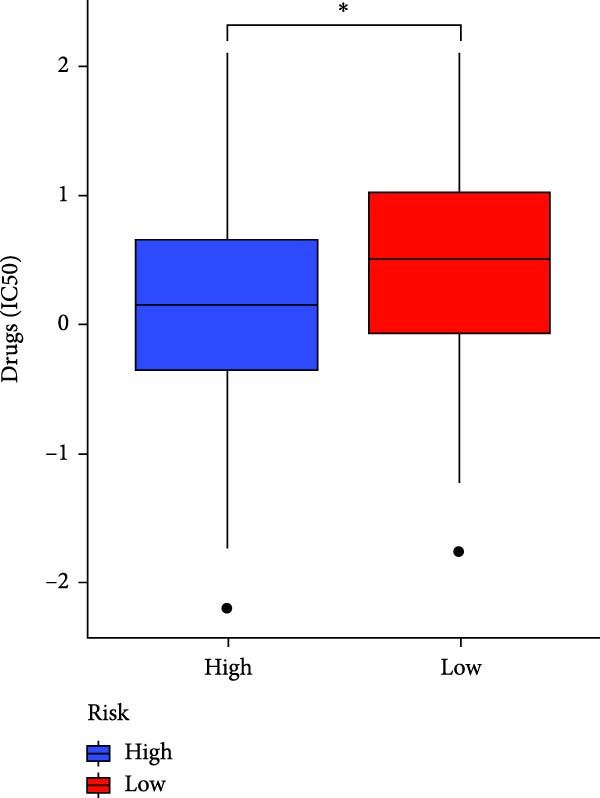
(G)

**Figure:**
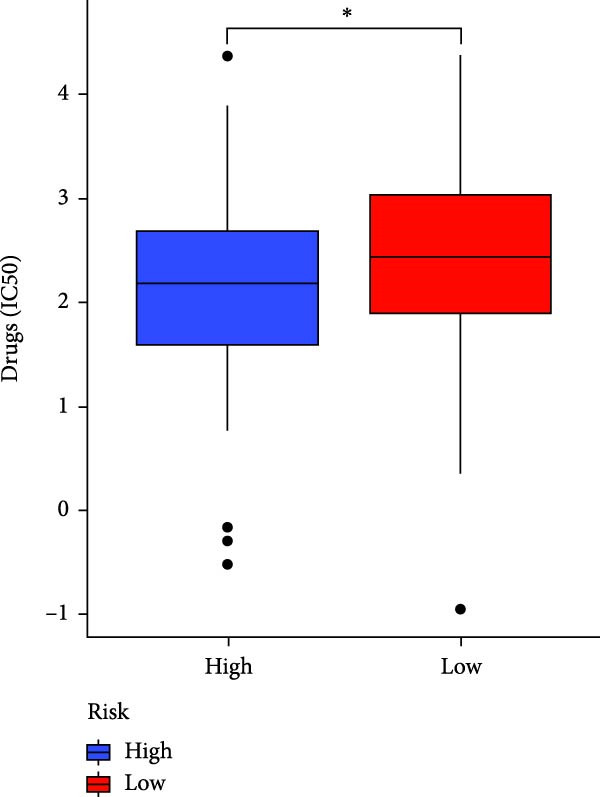
(H)

**Figure:**
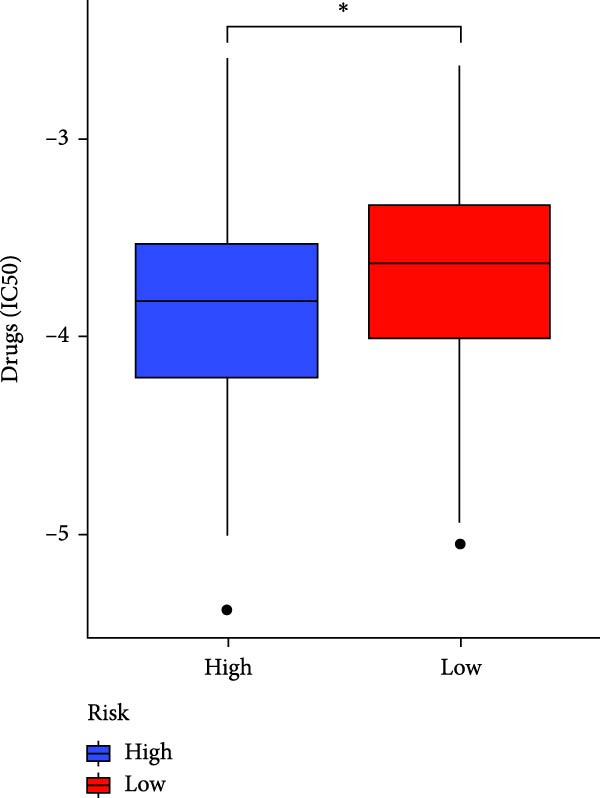
(I)

In addition, considering the widespread use of molecular targeted drugs in cancer treatment, we evaluated the predictive capability of the model for chemotherapy response using the “pRRophetic” R package based on the GDSC database. Six commonly used targeted drugs for advanced tumors were selected from the database: PARP inhibitor AZD2281, MEK1/2 inhibitors AZD6244 and PD0325901, selective PDK1 inhibitor BX795, MEK inhibitor RDEA119, and Aurora A inhibitor ZM447439. Patients were stratified into high‐risk and low‐risk groups based on the CAFs risk score model, and their drug sensitivity was assessed by predicted IC_50_ values. The results showed that the high‐risk group exhibited lower IC_50_ values for all these drugs compared to the low‐risk group (Figure 8D–I), highlighting that patients with high risk might be more affected by these specific therapies. In summary, the CAFs‐related risk model not only effectively predicts patient prognosis but also has potential utility in guiding personalized immunotherapy responses and targeted drug selection.

## 4. Discussion

CAFs, as an essential part of the TME, have been demonstrated to promote tumor growth through multiple mechanisms, including enhancing tumor angiogenesis and metastasis, fostering drug resistance, remodeling the extracellular matrix, and establishing an immunosuppressive microenvironment [32–35]. Hence, CAFs have long been recognized as indispensable pro‐tumor elements [36, 37]. Clavreul et al. isolated stromal cells associated with GBM that closely resemble CAFs in both phenotypic and functional characteristics, particularly exhibiting high expression levels of established CAFs markers [36, 38, 39]. Moreover, studies by Jain, Galbo, and colleagues have identified pro‐tumorigenic CAFs in GBM that drive invasion by inducing vascular hypertrophy and establishing perivascular niches [40, 41]. Unlike earlier research, we performed a systematic classification of CAFs subtypes based on scRNA‐seq data, revealing significant functional heterogeneity among these subpopulations.

Furthermore, accumulating evidence consistently underscores the prognostic significance of CAFs‐related genes and their secretory factors across multiple cancer types. For instance, elevated Transforming growth factor‐beta receptor type‐2 (TGFBR2) expression in breast CAFs is significantly associated with improved patient survival, and its independent prognostic value has been validated through both experimental studies and clinical trials [42]. In cholangiocarcinoma, specifically activated CAFs have been identified as indicators of unfavorable prognosis and compromised immunotherapy response [43]. Moreover, the six‐gene CAFs‐related risk signature developed by Yu et al. [25] has been verified for its predictive accuracy and reliability in hepatocellular carcinoma prognosis. Consistent with these observations, our predictive model identifies five CAFs subtypes with distinct prognostic implications in GBM. These findings not only expand the current understanding of CAFs‐mediated mechanisms but also highlight their translational potential for clinical application.

Using scRNA‐seq data, we performed a detailed classification of CAFs in GBM and identified several distinct CAFs subtypes. Notably, the CAF_1 subtype, primarily composed of malignant‐like cells, exhibited gene expression profiles highly enriched in multiple tumor‐associated signaling pathways, suggesting a critical role in promoting tumor invasion and metastasis. Furthermore, compared to normal tissues, the expression of CAFs subtype‐specific marker genes was markedly upregulated in GBM samples, and patients with high CAFs scores exhibited significantly poorer prognoses than those with low scores. These findings corroborate and extend previous studies, reinforcing the view that CAFs are key cellular components within the TME of GBM and are closely associated with tumor progression and clinical outcomes.

Building upon this, we identified six core CAFs‐associated genes (FAM241B, LSM2, IGFBP2, LOXL1, OSMR, STOX1) that were significantly correlated with patient prognosis. Based on these genes, we constructed a CAFs‐related risk score model that demonstrated robust prognostic performance across multiple independent datasets. The prognostic signature reliably categorized patients into distinct high‐ and low‐risk categories and showed strong associations with both overall survival and treatment response. Moreover, we developed a nomogram incorporating clinical parameters, which exhibited superior predictive accuracy and stability, outperforming the risk score model alone in identifying high‐risk patients. Collectively, our study underscores the critical role and prognostic significance of CAFs in TME, offering novel insights and theoretical foundations for risk stratification and personalized prognosis prediction in GBM.

In this study, we identified six key genes from CAFs‐related gene sets. Among these genes, the function of FAM241B remains unclear, it is localized to membrane structures such as the endoplasmic reticulum and shows differential expression in specific pathophysiological states like Parkinson’s disease models and lipid metabolism, suggesting it may be a conditionally activated gene [44–47]. Consistent with previous findings in GBM, hepatocellular carcinoma, and melanoma, our study confirms that elevated LSM2 expression signifies a poor prognosis and it may act as a common oncogenic factor across different cancer types [48–50]. Notably, OSMR, IGFBP2, and LOXL1 are highly expressed in multiple signaling pathways involved in tumor‐related biological processes such as apoptosis, inflammatory responses, EMT, and adherens junctions. These have been widely recognized as critical oncogenic drivers in various malignancies, participating in multiple pivotal stages of tumor development. In ovarian cancer, OSMR promotes tumor cell proliferation and metastasis by activating the STAT3 signaling pathway, and monoclonal antibodies targeting OSMR effectively suppressed cancer cell growth [51]. In GBM, OSMR cooperates with its ligand OSM to activate STAT3 signaling, inducing a mesenchymal‐like phenotype that enhances tumor invasiveness and treatment resistance [52]. IGFBP2 is consistently overexpressed in tissues and fluids from various solid tumors and is strongly associated with proliferation, migration, invasion, angiogenesis, and immune evasion. It plays a particularly important role in modulating M2 macrophage polarization and remodeling the immune microenvironment [53–59]. Similarly, LOXL1 promotes angiogenesis and EMT in GBM, osteosarcoma, prostate cancer, colon cancer, and breast cancer, making it a key driver of tumor dissemination and metastasis [60, 61]. Somatic SNV may also be implicated in GBM risk. Our study identified SNV in OSMR, STOX1, and LOXL1, though no significant co‐occurrence was found between these and other frequently mutated genes. Our findings suggest their potential involvement in disease development, indirectly supporting their association with tumor aggressiveness. Further analysis revealed strong correlations between these genes and poor clinical outcomes in GBM, indicating their pivotal roles in shaping the glioma microenvironment and driving tumor progression.

Immune evasion is one of the key mechanisms underlying tumor progression. CAFs contribute to the formation of an immunosuppressive TME by secreting a wide array of cytokines, growth factors, chemokines, exosomes, and other effector molecules, thereby modulating the functional status of immune cells and engaging in extensive cross‐talk with various components of the immune system, ultimately enabling tumor cells to evade immune surveillance [62, 63]. Our study further elucidates the intricate relationship between CAFs and the immune microenvironment. Immune infiltration analysis revealed a significant positive correlation between the expression levels of LOXL1, OSMR, and STOX1 and immune scores, suggesting that these genes may influence the TME and hold potential as therapeutic targets in GBM. Furthermore, we also found that immunosuppressive cells such as Th2 cells and M2 macrophages were significantly enriched in the high‐risk group. These findings suggest that a strongly immunosuppressive TME in these patients contributes to their poor prognosis.

Immunotherapy has shown promising efficacy in a variety of malignancies. However, the unique TME of GBM contributes to its extensive immunosuppressive mechanisms [64, 65]. T cell dysfunction further facilitates immune escape in glioma patients [66]. In this study, we validated the predictive capacity of our CAFs‐associated risk score model by comparing clinical outcomes in patients who received PD‐1/PD‐L1 immune checkpoint blockade therapy. The data suggested that patients who are at high risk exhibited stronger immune evasion potential and derived limited benefit from immunotherapy, whereas low‐risk patients showed more favorable clinical responses. These findings suggest that the proposed model can be used to detect patient subgroups that have a higher chance of benefiting from immunotherapeutic interventions, thereby offering clinical guidance for optimizing treatment strategies in GBM. More importantly, recent findings further suggest that specific CAFs subtypes may enhance therapeutic drug sensitivity. For instance, CD200‐positive CAFs significantly enhance the efficacy of the epidermal growth factor receptor (EGFR)‐tyrosine kinase inhibitor gefitinib against lung cancer cells [67]. Separately, it has been observed that CAFs can suppress the insulin‐like growth factor‐1 receptor (IGF‐1R) signaling through their distinct secretome, particularly via abundant IGFBP2, thereby sensitizing resistant tumor cells to conventional antitumor agents [68]. This finding aligns with our drug sensitivity analysis, in which the high‐risk group exhibited increased sensitivity to a range of targeted agents, including poly ADP‐ribose polymerase (PARP) inhibitors, Mitogen‐activated protein kinase kinase 1/2 (MEK1/2) inhibitors, the Pyruvate dehydrogenase kinase 1 (PDK1) inhibitor BX795, MEK inhibitors, and Aurora A inhibitors. These findings not only provide a theoretical basis for implementing precision therapy in high‐risk GBM patients but also offer novel insights for developing more personalized and targeted therapeutic strategies in the future.

This study, fully considering the high heterogeneity of CAFs, immune infiltration, the complex TME, and clinical characteristics, systematically characterized the subtypes of CAFs in GBM and their potential value in prognostic assessment, immune regulation, and drug sensitivity prediction using bioinformatic approaches. We developed and validated a novel prognostic model, which accurately predicts overall survival and immunotherapy response in GBM. The results provide direct evidence for risk stratification and precision management of GBM patients. However, several important limitations must be acknowledged. First, the findings primarily rely on retrospective bioinformatic analyses of public databases, and lack support from multi‐center, large‐sample clinical validation or experimental studies *in vitro* or *in vivo*, which may constrain the generalizability and robustness of the model. In particular, the expression patterns and prognostic relevance of key genes identified in this study—such as OSMR and LOXL1, as well as the less‐characterized FAM241B and LSM2—have not been verified. This gap limits the deeper understanding of their biological roles. Furthermore, the predictive model for immune response constructed in this study has not been systematically compared with existing mainstream immune scoring systems, and its clinical utility remains to be assessed in prospective trials. Second, although our work sheds light on the potential involvement of CAFs‐related genes in modulating the TME and tumor progression, the underlying molecular mechanisms—particularly how various CAFs subsets affect the TME and immunotherapy responses through these genes—remain elusive. Further functional studies, such as knockdown of OSMR or LOXL1 in GBM cell or stem cell models to examine effects on proliferation, apoptosis, and immune cell recruitment, or selective ablation of specific CAFs subpopulations in animal models to evaluate their causal roles in tumor progression and immune infiltration, are essential to confirm their functions. The current lack of such experimental validation constitutes a major limitation of this work. Finally, our analysis may be subject to inherent limitations of the data sources themselves, such as sample size, batch effects, and potential technical variations across different databases. These factors could introduce biases and affect the generalizability and stability of the constructed predictive model.

Notwithstanding these constraints, our study provides clear directions and reliable candidate targets for future research. Future studies will focus on validating these findings through multi‐center cohorts and delineating the molecular mechanisms through which key CAFs genes affect GBM outcomes, utilizing both *in vitro* and *in vivo* functional experiments. These efforts are expected to lay a solid foundation for the development of novel therapeutic strategies.

## 5. Conclusions

Our study has identified a novel risk scoring formula for GBM, which is based on six hub genes related to CAFs. This risk scoring model provides a comprehensive evaluation of the tumor immune microenvironment and risk characteristics of GBM patients. By constructing and validating this risk scoring formula, we have demonstrated its potential clinical application in predicting the prognosis of GBM patients and guiding personalized immunotherapy. Our research enhances the comprehension of the TME in GBM and provides fresh perspectives on the development of effective therapeutic strategies for this challenging disease. The robustness of our conclusions is supported by rigorous statistical analysis and validation using independent datasets, indicating the potential of our risk scoring model might serve as a beneficial tool in the clinical management of GBM.

## Conflicts of Interest

The authors declare that the research was conducted in the absence of any commercial or financial relationships that could be construed as a potential conflict of interest.

## Author Contributions

Conceptualization, Bingxuan Ren; Data curation, Hongyi Zhou, Xi Yang, Wen Zhao; Formal analysis, Jincheng Jiang, Xinchen Jiang; Funding acquisition, Bingxuan Ren; Investigation, Wen Zhao, Yingqi Huang, Kaixia Yang; Methodology, Zhixiang Zhang, Jincheng Jiang, Xinchen Jiang; Software, Yingqi Huang and Wen Zhao; Supervision, Bingxuan Ren and Kaixia Yang; Writing—review and editing, Hongyi Zhou, Xi Yang, Wen Zhao, Bingxuan Ren, Kaixia Yang. Hongyi Zhou, Xi Yang and Wen Zhao contributed equally to this work and should be listed as co‐first authors.

## Funding

Financial support was provided to the author(s) for conducting the research, writing, and/or publishing this article. This study was funded by grants from the Neurology Department of the National Key Clinical Speciality Construction Project (Grant No. 2023SZZ‐2), the Ningbo Major Research and Development Plan Project (Grant No. 2023Z196), the Project of Ningbo Leading Medical & Health Discipline (Grant No. 2022‐F05), and the Ningbo Medical and Health Brand Discipline (Grant No. PPXK2024‐01).

## Supporting Information

Additional supporting information can be found online in the Supporting Information section.

## Supporting information


**Supporting Information** Figure S1: (A) ssGSEA calculation sample enrichment grading the HALLMARK of a single cell. (B) GO‐KEGG analysis. (C) CAFs clusters ssGSEA rate of marker genes. (D) Patients’ prognosis of high and low CAFs score. (E) K‐M and ROC curves of the risk signature in GSE74187 cohort.

## Data Availability

The datasets relevant to the experiment were retrieved from the GEO database, TCGA database, and CGGA database and are available from the corresponding author on reasonable request.
