# Supplementary figures and images for: Glioblastoma Prognosis and Therapeutic Response Predicted by a Cancer‐Associated Fibroblasts Risk Score
Source: Mediators Inflamm. 2025 Dec 28;2025:4342537. doi: 10.1155/mi/4342537 (PMC12767406; doi:10.1155/mi/4342537)

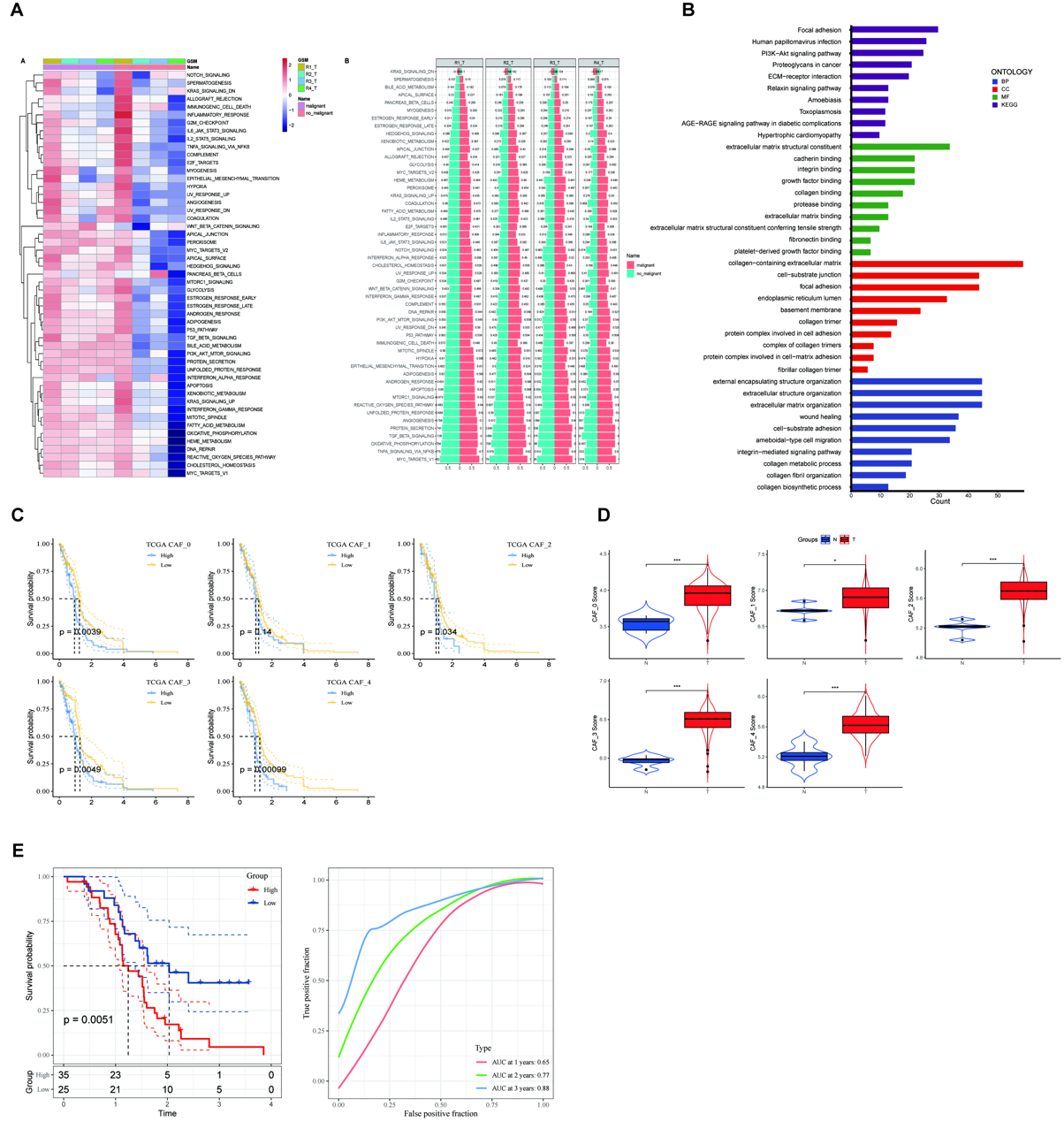

Supplement: Supplementary file 1 — Supporting Information Figure S1: (A) ssGSEA calculation sample enrichment grading the HALLMARK of a single cell. (B) GO‐KEGG analysis. (C) CAFs clusters ssGSEA rate of marker genes. (D) Patients’ prognosis of high and low CAFs score. (E) K‐M and ROC curves of the risk signature in GSE74187 cohort. [file MI-2025-4342537-s001.tif]
